# Recruiting Sexual and Gender Minority Veterans for Health Disparities Research: Recruitment Protocol of a Web-Based Prospective Cohort Study

**DOI:** 10.2196/43824

**Published:** 2023-10-02

**Authors:** Carolyn A Fan, Michelle Upham, Kristine Beaver, Krista Dashtestani, Malachi M Skiby, Kimberly Z Pentel, Isaac C Rhew, Michael R Kauth, Jillian C Shipherd, Debra Kaysen, Tracy Simpson, Keren Lehavot

**Affiliations:** 1 Department of Health Systems and Population Health University of Washington School of Public Health Seattle, WA United States; 2 Health Services Research & Development (HSR&D) Center of Innovation (COIN) VA Puget Sound Health Care System Seattle, WA United States; 3 Downtown Emergency Service Center (DESC) Seattle, WA United States; 4 Pacific Northwest Individual and Couple Therapy Seattle, WA United States; 5 Department of Psychiatry & Behavioral Sciences University of Washington School of Medicine Seattle, WA United States; 6 LGBTQ+ Health Program Office of Patient Care Services Veterans Health Administration Washington, DC United States; 7 South Central Mental Illness Research, Education and Clinical Center Michael E DeBakey VA Medical Center Houston, TX United States; 8 Department of Psychiatry Baylor College of Medicine Houston, TX United States; 9 National Center for Posttraumatic Stress Disorder (PTSD) VA Boston Healthcare System Boston, MA United States; 10 Boston University Chobanian & Avedisian School of Medicine Boston, MA United States; 11 Department of Psychiatry and Behavioral Sciences Stanford University Stanford, CA United States; 12 National Center for Posttraumatic Stress Disorder (PTSD) VA Palo Alto Health Care System Palo Alto, CA United States; 13 Center of Excellence in Substance Abuse Treatment and Education (CESATE) VA Puget Sound Health Care System Seattle, WA United States

**Keywords:** lesbian, gay, bisexual, transgender, queer, and other sexual and gender minority, LGBTQ+, veteran, recruitment, health disparities

## Abstract

**Background:**

The *Health for Every Veteran Study* is the first Veterans Health Administration–funded, nationwide study on lesbian, gay, bisexual, transgender, queer, and other sexual and gender minority (LGBTQ+) veterans’ health that relies exclusively on primary recruitment methods. This study aimed to recruit 1600 veterans with diverse sexual and gender identities to study the mental health and health risk behaviors of this population. A growing body of literature highlights the health inequities faced by LGBTQ+ veterans when compared with their heterosexual or cisgender peer groups. However, there is little to no guidance in the health disparities literature describing the recruitment of LGBTQ+ veterans.

**Objective:**

This paper provides an overview of the recruitment methodology of *Health for Every Veteran Study.* We describe the demographics of the enrolled cohort, challenges faced during recruitment, and considerations for recruiting LGBTQ+ veterans for health research.

**Methods:**

Recruitment for this study was conducted for 15 months, from September 2019 to December 2020, with the goal of enrolling 1600 veterans evenly split among 8 sexual orientation and gender identity subgroups: cisgender heterosexual women, cisgender lesbian women, cisgender bisexual women, cisgender heterosexual men, cisgender gay men, cisgender bisexual men, transgender women, and transgender men. Three primary recruitment methods were used: social media advertising predominantly through Facebook ads, outreach to community organizations serving veterans and LGBTQ+ individuals across the United States, and contracting with a research recruitment company, Trialfacts.

**Results:**

Of the 3535 participants screened, 1819 participants met the eligibility criteria, and 1062 completed the baseline survey to enroll. At baseline, 25.24% (268/1062) were recruited from Facebook ads, 40.49% (430/1062) from community outreach, and 34.27% (364/1062) from Trialfacts. Most subgroups neared the target enrollment goals, except for cisgender bisexual men, women, and transgender men. An exploratory group of nonbinary and genderqueer veterans and veterans with diverse gender identities was included in the study.

**Conclusions:**

All recruitment methods contributed to significant portions of the enrolled cohort, suggesting that a multipronged approach was a critical and successful strategy in our study of LGBTQ+ veterans. We discuss the strengths and challenges of all recruitment methods, including factors impacting recruitment such as the COVID-19 pandemic, negative comments on Facebook ads, congressional budget delays, and high-volume surges of heterosexual participants from community outreach. In addition, our subgroup stratification offers important disaggregated insights into the recruitment of specific LGBTQ+ subgroups. Finally, the web-based methodology offers important perspectives not only for reaching veterans outside of the Veterans Health Administration but also for research studies taking place in the COVID-19-impacted world. Overall, this study outlines useful recruitment methodologies and lessons learned to inform future research that seeks to recruit marginalized communities.

**International Registered Report Identifier (IRRID):**

DERR1-10.2196/43824

## Introduction

### Background

People who identify as lesbian, gay, bisexual, transgender, queer, and other sexual and gender minority (LGBTQ+) make up a sizable portion of US military service members and veteran populations. As of 2015, it has been estimated that 6.1% of current US military personnel have an LGBTQ+ identity [1]. This is a higher proportion than the overall US population; in the same year, 3.9% of US adults identified as part of the LGBTQ+ community [2]. Among male veterans, 1.9% were identified as gay, 2% as bisexual, and 0.5% as transgender. Among female veterans, 7% were identified as lesbian, 9.1% as bisexual, and 1.2% as transgender [1]. Both lesbian and bisexual women and transgender people have been shown to be overrepresented in military and veteran populations compared with the general population [3,4]. LGBTQ+ people join the military for a number of reasons, many of which may be similar to non-LGBTQ+ service members. However, LGBTQ+ people may be more likely to face violence, lack of acceptance from family, or socioeconomic challenges, leading them to choose the military as a career path [5,6].

These statistics are likely an underestimate, given the rising numbers of LGBTQ+ identification in the general population and the impact of homophobia, transphobia, and sexism in and out of the military [1,2,6]. In particular, “Don’t Ask, Don’t Tell,” established in 1994, prevented a generation of nonheterosexual service members from serving openly [6]. Over 14,000 service members were less-than-honorably discharged due to the policy until its repeal in 2011 [6]. LGBTQ+ veterans still face the aftereffects of the policy, continued attacks against transgender service members, and other forms of structural stigma [6]. Given the pressure to hide their identities, there could be more veterans identifying as LGBTQ+ than the current statistics describe.

A growing body of literature has highlighted the health inequities faced by LGBTQ+ veterans, both in comparison with their fellow veterans and the general population. LGBTQ+ veterans as a group demonstrate disparities in suicide mortality, depression, alcohol use, posttraumatic stress disorder, intimate partner violence including sexual violence, chronic obstructive pulmonary disease, asthma, stroke, and physical health [6-14]. Disparities can be found within specific LGBTQ+ subgroups. Research indicates that compared with non-LGBTQ+ female veterans, LGBTQ+ female veterans were more likely to feel unwelcome or unsafe within the Veterans Health Administration (VA) health care system and miss needed care [15]. A national study of transgender veterans found that 46% reported delaying medical care and that 36% delayed mental health care in the past year when they thought they needed it [16]. However, there has been limited research on more detailed subgroups. LGBTQ+ veterans are a diverse group across sexual orientation and gender identity, and research is still needed to identify additional physical and mental health disparities, as well as the causal pathways for these inequities. In particular, structural stigma in the form of homophobic and transphobic policies, practices, and norms plays a significant role in causing health disparities among LGBTQ+ populations [17].

Therefore, it is crucial to better understand how to reach and recruit LGBTQ+ veterans in health research. As with other marginalized populations, LGBTQ+ veterans’ unique social contexts and lived experiences are shaped by their intersecting identities. However, there is little to no guidance in the health disparities literature describing the recruitment of LGBTQ+ veterans.

VA electronic medical records (EMRs) do not yet have readily accessible self-identified fields for sexual orientation or gender identity [18]. Although this is starting to change, it has been difficult for researchers studying LGBTQ+ veterans to monitor target populations for research or clinical care purposes through administrative records, as is often done for other groups, such as women [18]. Past research with transgender veterans has relied on the International Classification of Disease, 9th and 10th Revisions diagnosis codes in EMR data, rather than self-identified gender [19-21]. Such data can be obtained through VA EMRs [22]. Although these diagnostic codes have shown concordance with self-identification [20], they also rely on terms or diagnoses that may be outdated, stigmatizing, or misaligned with how transgender patients identify [23]. As such, veterans may choose not to disclose their gender identity to their VA providers. In addition, these diagnosis codes are specific to gender identity, meaning that sexual minority veterans cannot be identified using EMR data. Moreover, recruitment methods that rely solely on VA EMR data exclude veterans who are not connected with VA care. Veterans who were dishonorably discharged are not eligible for VA health care benefits, and others may choose not to enroll. LGBTQ+ veterans may decide not to engage with VA because of their previous experiences with discrimination in the military, VA, or other health care settings. Anticipation or fear of such experiences may also prevent LGBTQ+ veterans from connecting with the VA. As such, EMR-based recruitment methods for research are not yet sufficiently inclusive for the recruitment of a diverse sample of LGBTQ+ veterans.

In civilian communities, the recruitment of LGBTQ+ populations for health research often occurs through community-based sites [24] and social media [25]. Historically, emphasis has been placed on men who have sex with men, with less information on the recruitment of sexual minority women and other groups [24,26]. For instance, a systematic review of the psychology literature on LGBTQ+ populations found that sexual minority women comprised only 4.8% of the study samples [26]. Therefore, the civilian LGBTQ+ health research recruitment literature may also have limited applicability for researchers hoping to recruit a wide range of LGBTQ+ veterans in health research. Literature on the web-based recruitment of gay and bisexual men describes the use of social media websites (eg, Facebook), gay news sites, and dating and sexual networking mobile apps [25]. Web-based recruitment has also been paired with in-person community-based recruitment at places targeted toward gay and bisexual men [25]. The literature on recruiting transgender people into health research also describes the use of social media ads, in addition to partnering with transgender individuals with similar lived experiences as the desired study population to conduct outreach [27,28].

Finally, race and ethnicity are other dimensions of identity that are important for researching the health of LGBTQ+ veterans. Little research exists on the recruitment of participants across the intersection of these identities (race, ethnicity, sexual orientation, gender identity, and veteran status). Structural racism and the ways in which it intersects with homophobia, transphobia, and sexism affect health [29-31]. As such, it is important to recruit LGBTQ+ racial and ethnic minority veterans into health disparities research, who have lived experiences with these intersectional forms of discrimination both in and out of military service.

Thus, gaps remain in the health research literature regarding the recruitment of individuals at the intersection of LGBTQ+ and veteran identities. Further knowledge is needed to effectively reach veterans in and out of the VA and across sexual orientation and gender identity; this paper addresses these gaps. To our knowledge, no previous study has focused on the recruitment of people who identify as LGBTQ+ *and* as veterans. In addition, we have described the efforts to recruit LGBTQ+ racial and ethnic minority veterans. By more effectively recruiting a diverse group of LGBTQ+ veterans, researchers can conduct health research that informs targeted interventions, treatments, and prevention strategies tailored to the specific needs of this population.

### Objectives

The *Health for Every Veteran Study* is one such study that aims to better understand the health of LGBTQ+ veterans (VA Health Services Research & Development Study, Investigator Initiated Research 17-089). This is the first VA-funded nationwide study on LGBTQ+ veteran health that relies exclusively on primary recruitment methods, meaning that the data were collected directly from individuals prospectively. The *Health for Every Veteran Study* offers a unique opportunity to learn more about the recruitment of LGBTQ+ veterans for health research, particularly through web-based methods.

This paper describes the following: (1) the recruitment methodology of the *Health for Every Veteran Study*, (2) demographic characteristics of the enrolled cohort, (3) challenges faced during recruitment, and (4) considerations for recruiting LGBTQ+ veterans in health research.

## Methods

### Overall Recruitment Methodology

The *Health for Every Veteran Study* is a web-based, national, VA-funded, prospective cohort study on health disparities among LGBTQ+ veterans. The objectives of this study are as follows: (1) identify the extent of sexual orientation and gender identity disparities in mental health problems (eg, depression, posttraumatic stress disorder, anxiety, nonimminent suicidal ideation or attempt) and health risk behaviors (eg, alcohol misuse and smoking) among veterans; (2) examine risk and protective factors associated with these outcomes guided by a conceptual model informed by the minority stress theory and the self-medication hypothesis; and (3) assess LGBTQ+ veterans’ experiences with and preferences for treatment, including VA health care use, barriers to access, and preferences for tailored care. This study aims to include a wide range of veterans, including those who are and are not enrolled in VA health care.

Recruitment was conducted over 15 months, from September 2019 to December 2020, with the goal of enrolling 1600 veterans evenly split among 8 subgroups (200 veterans/subgroup): cisgender heterosexual women, cisgender lesbian women, cisgender bisexual women, cisgender heterosexual men, cisgender gay men, cisgender bisexual men, transgender women, and transgender men. Male and female heterosexual cisgender participants are included as comparison groups to assess the disparities. The transgender groups include veterans of all sexual orientations because splitting those groups further by sexual orientation was not seen as feasible due to sample size concerns, budget, and timeline. Although not explicitly targeted in recruitment efforts, several interested participants indicated a gender identity not represented by the above groups. As such, an exploratory subgroup comprising nonbinary and genderqueer veterans and veterans with other gender-diverse identities was added after the recruitment had begun.

Three primary recruitment methods were used in this study: (1) social media advertising conducted by staff, predominantly through Facebook ads; (2) outreach to community organizations serving veterans and LGBTQ+ individuals across the United States; and (3) contracting with a research recruitment company, Trialfacts.

### Social Media Advertising

On the basis of previous research on recruitment experience using social media to reach female and transgender veterans [32,33], the study team created several ad sets for use on social media platforms. Facebook was the primary social media platform used; Google and Instagram ads were tested for 6 and 12 weeks, respectively, but were discontinued for cost-effectiveness reasons.

**Figure 1:**
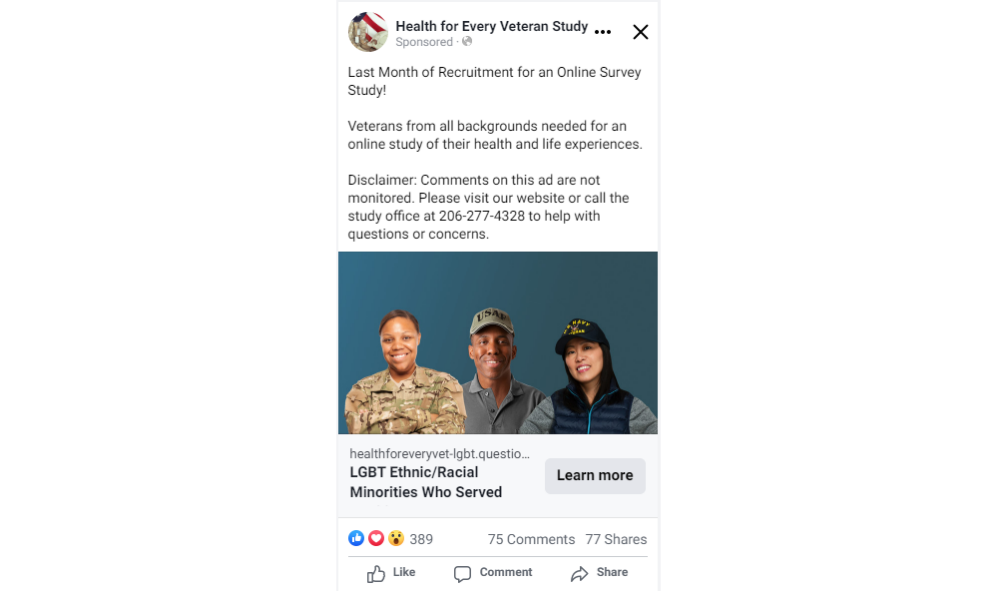
Facebook ad created by study staff for the *Health for Every Veteran Study* aimed toward lesbian, gay, bisexual, transgender, queer, and other sexual and gender minority veterans who also identify as racial and ethnic minorities.

**Figure 2:**
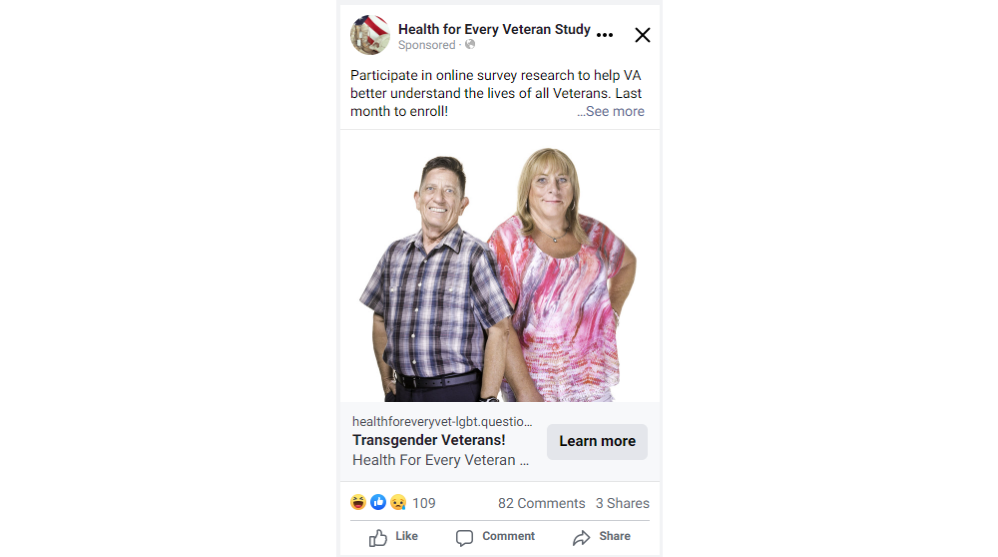
Facebook ad aimed toward transgender veterans created by study staff for the *Health for Every Veteran Study*.

**Figure 3:**
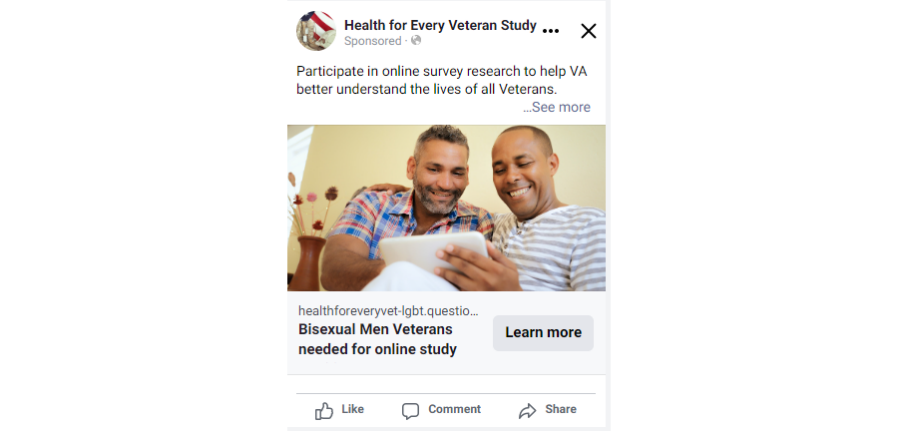
Facebook ad aimed toward bisexual male veterans created by study staff for the *Health for Every Veteran Study*.

**Figure 4:**
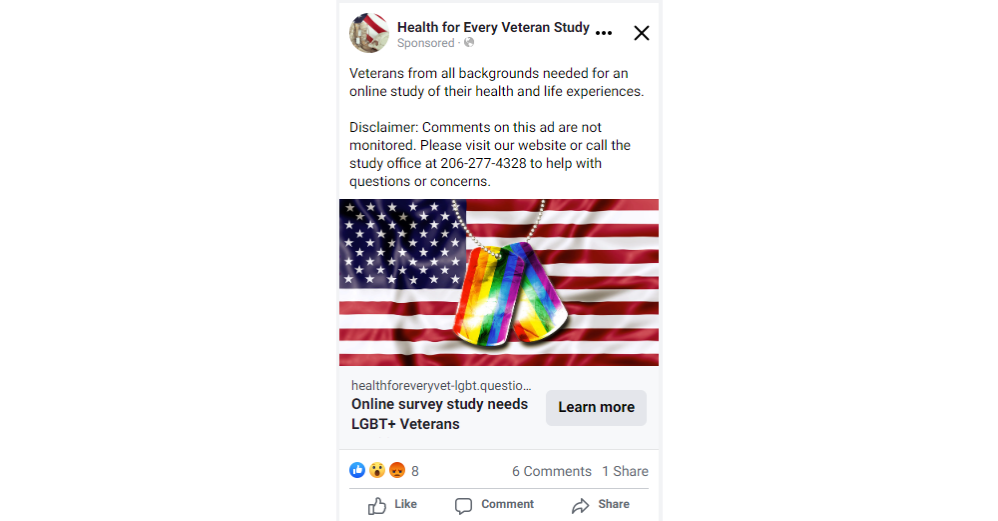
Facebook ad aimed toward lesbian, gay, bisexual, transgender, queer, and other sexual and gender minority veterans created by study staff for the *Health for Every Veteran Study*.

Several Facebook ad sets were created by the study staff and approved by the VA Puget Sound Institutional Review Board (IRB), each with targeted images and language carefully selected to reflect the various recruitment subgroups (Figures 1-4). The staff selected photos for ads from Adobe stock photos, and the VA Medical Media Department assisted in editing and tailoring images for certain subgroups, such as LGBTQ+ racial and ethnic minority veterans.

Although the Facebook policy does not allow for the explicit targeting of users by sexual orientation or relationship status, other audience parameters were used to reach the target population. For instance, gender, military service, and affiliation with LGBTQ+ and military organizations were used to further target the individuals through ads.

The most successful ads, determined by the number of views and clicks, were shown more often as part of Facebook’s ad-optimization algorithm. The use of separate ad sets also allowed the study staff to fine-tune the budget on a given ad set to adjust to the study’s changing recruitment goals across subgroups. This gave staff the opportunity to prioritize a larger percentage of the study’s budget toward harder-to-reach subgroups such as bisexual veterans. Geographic parameters of audience viewers were also adjusted to improve representativeness across geographic regions (eg, limiting ad audiences to underrepresented states in the study sample).

Facebook ads were initially placed as right-column ads and were later expanded to newsfeed ad placements to reach both desktop and mobile app users. Due to the VA Puget Sound IRB policy, direct interactions with veterans on social media were prohibited. Commenting cannot be disabled on newsfeed ads; therefore, an IRB–approved disclaimer was included on those ads to inform viewers that all questions or concerns should be relayed to the study staff directly by phone. Comment sections on all ads were monitored for influxes of negative and derogatory comments. Filtering tools available on Facebook were used, and manual staff reviews were conducted daily to block or hide all comments.

### Community Organization Outreach

The community organization outreach recruitment method served two purposes: (1) to reach veterans who do not regularly use social media and (2) to establish more trust with populations who may otherwise not feel comfortable participating in research. For this recruitment method, the study staff created a database of community organizations serving veterans and LGBTQ+ individuals across the United States. Community organizations were identified through internet searches by the study staff, and their information was added to the study recruitment database. These community organizations included nonprofit, grassroots organizations and other community-based organizations at the national, state, and local levels. For instance, organizations that shared the study advertisements included the Modern Military Association of America, a nonprofit dedicated to LGBTQ+ service members, veterans, and their families, and SPART*A, an organization of transgender (including nonbinary and gender nonconforming) service members and veterans. Local organizations across all 50 states were included, as were organizations representing various subgroups (eg, transgender groups and bisexual groups). The search ultimately expanded to LGBTQ+ and veteran groups at universities, community colleges, and large employers (eg, Microsoft, Starbucks, and health care companies).

The size, demographics (age, race, ethnicity, sexual orientation, gender identity, etc), and locations of these organizations were intentionally diversified. In particular, a concerted effort was made to recruit veterans who identified as racial and ethnic minority veterans in the study. Organizations serving racial and ethnic minority veterans, whether LGBTQ+ focused or not, were included in the internet search and contact process. Nonveteran groups serving LGBTQ+ veterans, racial and ethnic minority veterans, and LGBTQ+ racial and ethnic minority veterans were also included in recruitment efforts in recognition that civilian groups may also include veteran members.

The final database included 2942 community organizations. Staff sent initial emails to the main contact listed for each of these organizations by using “mail merge,” which is an email feature that allows users to send personalized emails to large batches of recipients by uploading spreadsheets with appropriate contact information. After the first email contact, up to 4 follow-up reminders were sent via email if there was no initial response. Those who agreed to participate were recontacted at future time points and asked to reshare study information and tailored ads.

The initial email message shared background information about the study, with a request to directly share study ads and contact information with members via email distribution lists, bulletin boards, the organization’s social media platforms, or other communication methods regarding their preferences. The email messages and ads were tailored to the community organization’s membership base. For instance, the language of a contact email to an organization serving transgender veterans was adjusted to highlight how the study sought to better understand the experiences of transgender veterans, and ads designed for transgender veterans were attached with the email.

Ultimately, 10.47% (308/2942) of the organizations responded to the study staff and agreed to share the study information with their membership bases. Organizations that assisted with the recruitment were publicly acknowledged on the study website with their permission. After the completion of the full study, the participating organizations will receive an update on the study findings.

Given the nature of community outreach, there were, at times, high-volume surges of interested participants from a single organization. These surges occurred after large organizations sent out study information to their membership bases, resulting in many members completing the web-based screener. All high-volume surges were manually reviewed, and screening decisions were discussed by the study team and documented to ensure that the study sample was not biased by a single organization or geographic region. When surges occurred, the study’s current enrollment profile was reviewed, and subgroups requiring additional participants were prioritized. Oftentimes, surges were comprised of predominantly White cisgender male heterosexual veterans. Therefore, LGBTQ+, female, and racial and ethnic minority veterans were prioritized for screening.

### Trialfacts

In the first 9 months of recruitment, enrollment numbers had not yet reached the target goals. Therefore, in May 2020, the study team decided to contract with an outside company to bolster its recruitment efforts by the study team. Trialfacts, a company that specializes in recruitment services for the clinical trial industry [34], was chosen due to its due diligence process and prior success with recruiting veteran populations.

Trialfacts was paid to use Facebook advertising to reach potential participants, as well as reaching out to a pool of participants from previous Trialfacts recruitment efforts in their internal database. These Facebook ads were run by Trialfacts employees, who were able to use their marketing and social media expertise to monitor and optimize ads consistently. The study team shared ad images and language that were shown to be successful in the study team’s own social media ads, and Trialfacts also created their own IRB–approved ads with staff input and guidance.

Typically, Trialfacts guarantees a minimum number of participant referrals when contracting with study teams. However, Trialfacts had no prior experience of specifically recruiting LGBTQ+ veterans. As a result, Trialfacts proposed approaching this study as a pilot program. As such, the study team worked closely with the Trialfacts staff during the recruitment process. In addition to cocreating ads, the eligibility of Trialfacts referrals was relayed back to Trialfacts on a regular basis to help Trialfacts fine-tune its recruitment methods.

Trialfacts helped recruit participants across all study subgroups, although certain subgroups were prioritized in the study budget based on recruitment needs. In particular, the study team requested Trialfacts to focus on the recruitment of bisexual female and male, transgender male, and racial and ethnic minority veterans. In addition, Trialfacts used geotargeting and other advertising strategies to increase overall demographic diversity, with the goal of increasing the national representativeness of the enrolled sample.

### Screening and Enrollment

**Figure 5:**
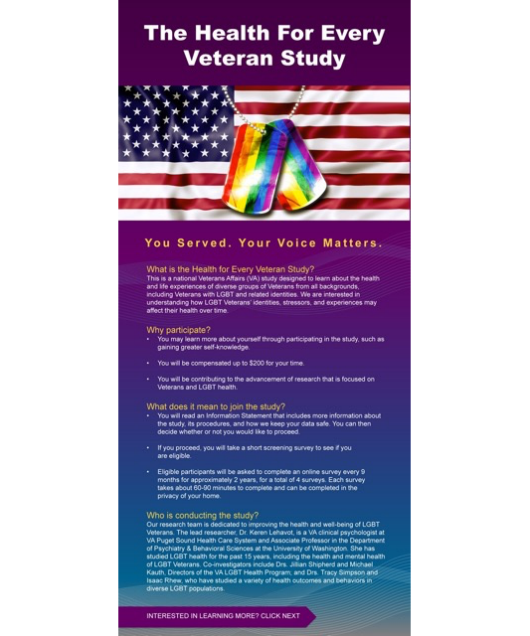
*Health for Every Veteran Study* information landing page for lesbian, gay, bisexual, transgender, queer, and other sexual and gender minority populations.

**Figure 6:**
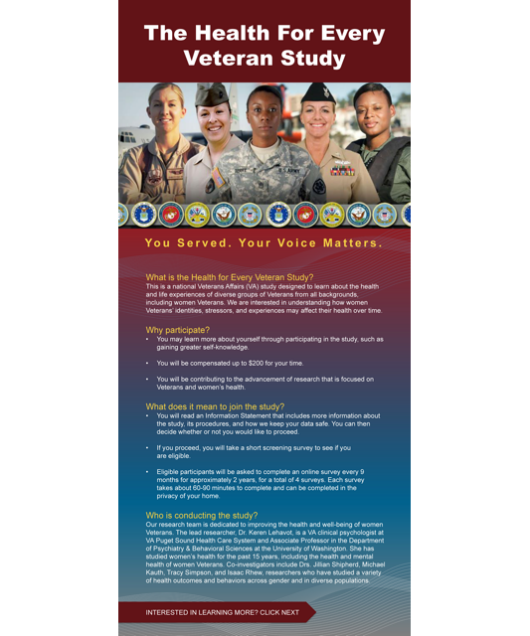
*Health for Every Veteran Study* information landing page for female veteran populations.

**Figure 7:**
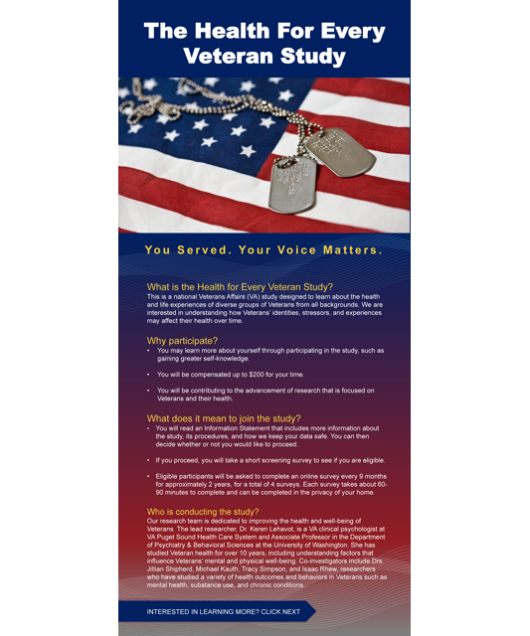
*Health for Every Veteran Study* information landing page for general veteran populations.

Interested participants who were recruited via staff social media ads, community outreach, and Trialfacts were directed to an information landing page hosted by QuestionPro. These landing pages were tailored based on the ads on which the interested participant clicked; unique language and images were displayed based on the tailored subgroup for that ad. For example, ads for LGBTQ+, female, and general veteran audiences would link to LGBTQ+, women, and general landing pages, respectively (Figures 5-7). These landing pages contained relevant information about the *Health for Every Veteran Study* for interested participants, including benefits of participation, number of follow-ups (every 9 mo for 27 mo), and a description of the study team.

If participants indicated that they were interested in participating, they would then be directed to the study information statement, screening consent form, and the study screener (which were all the same across participants and hosted in QuestionPro) to determine eligibility. The eligibility criteria mandated that the veteran must be aged ≥18 years; be currently nonincarcerated; and have prior service in the US military, US residence, routine internet access, valid contact information, and willingness to answer questions about key demographics. Individuals recruited through Trialfacts underwent the same screening procedures, although the landing pages and study screeners were housed and conducted by Trialfacts.

All prospective participants underwent a manual review to gauge veteran “insider knowledge” to ensure authentic veteran status. In this manual review, the study staff reviewed responses to questions regarding military branches, job fields and acronyms, and rank to determine the validity of the prospective participant’s veteran status. In addition, a statement was also included on the screener as a deterrent to falsifying responses saying the following: “Per the Stolen Valor Act of 2013, it is a federal crime to obtain money or other benefits by falsely representing prior military service.” Additional screening precautions are detailed in our paper elaborating the baseline results [35]. Those who did not pass the screener or manual review were deemed ineligible and provided with a link to a detailed resource page that included contact information for emergency crisis services, mental health services, sexual assault and domestic violence support, substance use support, organizations serving specific populations such as female and LGBTQ+ veterans, and information on VA benefits.

Eligible participants were emailed a link to the 60- to 90-minute web-based baseline survey to be completed on a website developed by the VA Cooperative Studies Program. If completed, the participants were considered to be enrolled and compensated US $30. Additional compensation was provided for follow-up surveys as follows: US $35 for the 9-month follow-up, US $40 for the 18-month follow-up, US $45 for the 27-month follow-up, and an additional US $50 bonus for the completion of all surveys. As such, participants could earn up to US $200 for their time. According to the VA policy, payments were issued by the Department of Treasury via physical checks or electronic fund transfers.

Weekly reporting of screening and enrollment was conducted by the study team to track the recruitment process. Screened and enrolled participants were examined by the subgroup and demographics to better understand the enrollment needs and tailor recruitment strategies across all recruitment methods. Demographic characteristics from national research surveys, such as the Behavioral Risk Factor Surveillance Survey, National Epidemiologic Survey on Alcohol and Related Conditions-III, and Millennium Cohort, were reviewed and compared by demographics (ie, age, race, marital status, education, income, and geographic region) to improve national representativeness. Additional strategies were used to optimize validity, such as not including study compensation amounts in advertising and monitoring for duplicate identifying information.

### Ethical Considerations

This study was approved by the VA Puget Sound Health Care System IRB (01672) and granted a waiver for the documentation of informed consent. Potential participants were provided with an information statement comprehensively describing the nature of the study as well as its risks and benefits upon visiting the study website. All study data in this paper have been deidentified to protect the privacy and confidentiality of the participants.

## Results

**Figure 8:**
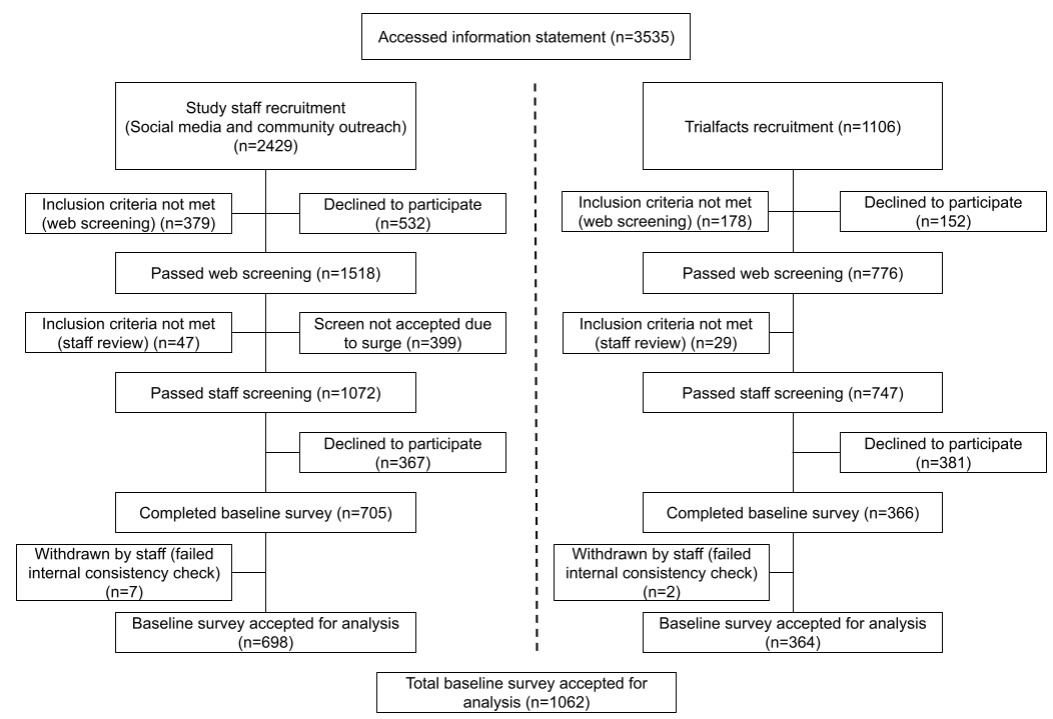
*Health for Every Veteran Study* CONSORT (Consolidated Standards of Reporting Trials) diagram demonstrating study staff recruitment and Trialfacts recruitment.

Enrollment in the *Health for Every Veteran Study* concluded in December 2020. A total of 3535 participants accessed the study information statement to begin the screening process, and 1819 (51.46%) participants passed screening; of these 1819 participants, 1062 (58.38%) completed the baseline survey to enroll. The CONSORT (Consolidated Standards of Reporting Trials) diagram [36] demonstrates the flow of recruitment, from screening to enrollment, divided across study staff recruitment methods (ie, social media advertising and community outreach) and Trialfacts recruitment (Figure 8). All descriptive results were calculated using R (R Foundation for Statistical Computing) [37].

Of the enrolled participants (n=1062), 25.24% (n=268) were recruited from staff Facebook ads, 40.49% (n=430) from community outreach, and 34.27% (n=364) from Trialfacts. Trialfacts proved to be instrumental for the study, providing one-third (364/1062, 34.27%) of the participants despite being initiated 9 months after the recruitment had begun. Notably, all 3 methods of recruitment contributed to substantial portions of the enrolled cohort, indicating that a multipronged approach was a critical and successful strategy for the recruitment of hard-to-reach populations in our study of LGBTQ+ veterans.

Of the 8 subgroups targeted for recruitment, 5 subgroups neared the target recruitment goal of 200 participants per subgroup: cisgender gay men, cisgender heterosexual women, cisgender heterosexual men, cisgender lesbian women, and transgender women. However, cisgender bisexual men, cisgender bisexual women, and transgender men did not reach recruitment goals. Table 1 describes the enrollment by subgroup, including the percentage of the target enrolled. Of note is the subgroup of gender-diverse veterans; although they were not an a priori recruitment group, they were enrolled as an exploratory group after recruitment began to be inclusive of the experiences of veterans not encompassed by the other a priori subgroups.

Table 2 demonstrates the demographic breakdown of the study sample by recruitment method. Various recruitment methods were effective at recruiting different subgroups; however, it should be noted that the methods were tailored to target specific subgroups based on needs at various points of the recruitment period. For instance, because community outreach methods were extremely successful in recruiting cisgender heterosexual male and cisgender female veterans (at times resulting in recruitment surges; see the *Discussion* section), only a small fraction of Trialfacts ads focused on these populations. Instead, Trialfacts purposefully focused on the recruitment of cisgender bisexual men, cisgender bisexual women, and transgender men. This proved critical; 68% (42/62) of cisgender bisexual men were recruited through Trialfacts, compared with 11% (7/62) through Facebook and 21% (13/62) through community outreach (Table 2). Similarly, Trialfacts recruited 48% (29/61) of cisgender bisexual women and 49% (22/45) of transgender men (Table 2). To speak to the broader demographic representativeness of participants gleaned from each recruitment method, information on age, race, ethnicity, education, income, and marital status of the enrolled sample by recruitment method is also included in Table 2.

**Table 1:** Enrollment by recruitment subgroup.

Gender identity and gender and sexual orientation		Target enrollment (N=1600), n (%)	Enrolled (n=1062, 66.4%), n (%)^a^
**Men**			
	Cisgender gay men	200 (12.5)	189 (94.5)
	Cisgender bisexual men	200 (12.5)	62 (31)
	Transgender men	200 (12.5)	45 (22.5)
	Cisgender heterosexual men	200 (12.5)	169 (84.5)
**Women**			
	Cisgender lesbian women	200 (12.5)	154 (77)
	Cisgender bisexual women	200 (12.5)	61 (30.5)
	Transgender women	200 (12.5)	144 (72)
	Cisgender heterosexual women	200 (12.5)	172 (86)
Gender-diverse (eg, nonbinary, gender nonconforming, and genderqueer) individuals		N/A^b^	66 (33)

^a^Percentage of the target enrollment achieved (n=200).

^b^N/A: not applicable; not an a priori group.

**Table 2:** Demographics of enrolled sample by recruitment source.

		Facebook (n=268, 25.2%), n (%)^a^	Community outreach (n=430, 40.5%), n (%)^a^	Trialfacts (n=364, 34.3%), n (%)^a^	Overall (N=1062), n (%)^b^
**Study subgroup**					
	Cisgender heterosexual men	43 (25.4)	111 (65.7)	15 (8.9)	169 (15.9)
	Cisgender gay men	49 (25.9)	64 (33.9)	76 (40.2)	189 (17.8)
	Cisgender bisexual men	7 (11.3)	13 (21)	42 (67.7)	62 (5.8)
	Transgender men	8 (17.8)	15 (33.3)	22 (48.9)	45 (4.2)
	Cisgender heterosexual women	68 (39.5)	96 (55.8)	8 (4.7)	172 (16.2)
	Cisgender lesbian women	34 (22.1)	53 (34.4)	67 (43.5)	154 (14.5)
	Cisgender bisexual women	18 (29.5)	14 (23)	29 (47.5)	61 (5.7)
	Transgender women	34 (23.6)	44 (30.6)	66 (45.8)	144 (13.6)
	Gender-diverse individuals	7 (10.6)	20 (30.3)	39 (59.1)	66 (6.2)
**Age group (years)**					
	20-25	0 (0)	10 (40)	15 (60)	25 (2.4)
	26-35	38 (21.8)	46 (26.4)	90 (51.7)	174 (16.4)
	36-45	49 (22.9)	74 (34.6)	91 (42.5)	214 (20.2)
	46-55	57 (27.3)	75 (35.9)	77 (36.8)	209 (19.7)
	56-65	64 (28.6)	94 (42)	66 (29.5)	224 (21.1)
	>65	60 (27.8)	131 (60.6)	25 (11.6)	216 (20.3)
**Race and ethnicity**					
	Black and non-Hispanic	18 (36.7)	25 (51)	6 (12.2)	49 (4.6)
	Hispanic	20 (22)	35 (38.5)	36 (39.6)	91 (8.6)
	White, non-Hispanic	212 (25.3)	333 (39.8)	292 (34.9)	837 (78.8)
	Other race, non-Hispanic	5 (18.2)	12 (54.5)	5 (22.7)	22 (2.1)
	Multiracial, non-Hispanic	13 (20.6)	25 (39.7)	25 (39.7)	63 (5.9)
**Education**					
	12th grade	16 (34)	14 (29.8)	17 (36.2)	47 (4.4)
	Vocational or training >12th grade	15 (35.7)	10 (23.8)	17 (40.5)	42 (4)
	Some college	87 (24)	127 (35.1)	148 (40.9)	362 (34.1)
	College graduate	47 (20.7)	91 (40.1)	89 (39.2)	227 (21.4)
	Some graduate or professional	18 (20.7)	43 (49.4)	26 (29.9)	87 (8.2)
	Master’s degree	67 (28.3)	112 (47.3)	58 (24.5)	237 (22.3)
	Doctoral degree	17 (29.3)	32 (55.2)	9 (15.5)	58 (5.5)
	Missing	1 (50)	1 (50)	0 (0)	2 (0.2)
**Income (US $)**					
	<15,000	13 (25)	13 (25)	26 (50)	52 (4.9)
	15,000 to ≤20,000	10 (29.4)	11 (32.4)	13 (38.2)	34 (3.2)
	20,000 to ≤25,000	12 (25)	16 (33.3)	20 (41.7)	48 (4.5)
	25,000 to ≤30,000	8 (18.6)	14 (32.6)	21 (48.8)	43 (4)
	30,000 to ≤40,000	22 (22.4)	38 (38.8)	38 (38.8)	98 (9.2)
	40,000 to ≤50,000	34 (31.5)	36 (33.3)	38 (35.2)	108 (10.2)
	50,000 to ≤60,000	21 (21.4)	37 (37.8)	40 (40.8)	98 (9.2)
	60,000 to ≤70,000	18 (21.4)	32 (38.1)	34 (40.5)	84 (7.9)
	70,000 to ≤80,000	27 (26.2)	42 (40.8)	34 (33)	103 (9.7)
	80,000 to ≤90,000	16 (26.2)	28 (45.9)	17 (27.9)	61 (5.7)
	90,000 to ≤100,000	14 (29.8)	19 (40.4)	14 (29.8)	47 (4.4)
	≥100,000	71 (25.5)	138 (49.6)	69 (24.8)	278 (26.2)
	Missing	2 (25)	6 (75)	0 (0)	8 (0.8)
**Marital status**					
	Married or domestic partnership	141 (25.9)	240 (44)	164 (30.1)	545 (51.3)
	Never married	53 (25.6)	76 (36.7)	78 (37.7)	207 (19.5)
	Separated	5 (15.2)	6 (18.2)	22 (66.7)	33 (3.1)
	Divorced	58 (25.6)	85 (37.4)	84 (37)	227 (21.4)
	Widowed	9 (27.3)	14 (42.4)	10 (30.3)	33 (3.1)
	Other	2 (12.5)	8 (50)	6 (37.5)	16 (1.5)
	Missing	0 (0)	1 (100)	0 (0)	1 (0.1)
**Region**					
	Northeast	30 (25.6)	52 (44.4)	35 (29.9)	117 (11)
	South	109 (25.1)	165 (37.9)	161 (37)	435 (41)
	Midwest	52 (24.8)	81 (38.6)	77 (36.7)	210 (19.8)
	West	76 (25.5)	131 (44)	91 (30.5)	298 (28.1)

^a^Percentage of subgroup enrollment by recruitment source.

^b^Percentage of overall enrollment by subgroup.

## Discussion

### Recruitment Methods

*The Health for Every Veteran Study* is the first study to specifically recruit a diverse sample of LGBTQ+ and heterosexual cisgender veterans nationwide to examine health disparities among these subgroups of veterans. Recruitment results indicated that all 3 recruitment methods, namely, Facebook ads, community outreach, and contracting with a recruitment company, were crucial for the recruitment and enrollment success. Each method contributed to at least a quarter of the enrolled participants.

In addition to the number of participants recruited, the study staff found unique benefits and costs for each method. Because recruitment strategies were continuously adjusted to best meet the study needs, all final recruitment numbers (both overall and for specific subgroups) should be interpreted within the context of this study’s time period, population, and aims. However, lessons learned from the study’s recruitment methodology and strategies can be applied to a wide range of researchers who work with hard-to-reach populations. To capture this, Table 3 describes various strengths and challenges of all 3 recruitment methods based on the study team’s experience.

In particular, a company that specializes in recruitment strategies for research may be an attractive option for researchers with limited abilities or staff resources to manage or optimize social media ads. Despite launching this partnership 9 months after recruitment had begun, Trialfacts brought in 34% of the enrolled participants. Using Trialfacts at the start of the study recruitment period may have allowed the study to achieve full enrollment. However, researchers should consider whether they wish to have a single source for recruitment or whether a diversified recruitment strategy is desired. Depending on the study’s aims and the desired target population, different strategies and methods may be beneficial.

Notably, each VA health care system has a designated LGBTQ+ Veteran Care Coordinator (VCC). VCCs serve as the point persons for LGBTQ+ veteran affairs at their respective sites. However, we intentionally chose not to engage with VCCs for recruitment to reduce bias that would have been introduced had we recruited veterans who were already engaged in VA services. For other researchers, connecting with VCCs for recruitment may be an ideal option for the recruitment of LGBTQ+ veterans.

**Table 3:** Strengths and challenges of study recruitment methods.

Recruitment method	Strengths	Challenges
Facebook ads	Reach those not affiliated with the VA^a^ or connect with wider audience Can target audience by some parameters to optimize reach Can adjust ads and targeting in real time, improves cost-efficiency, and can follow recruitment needs for specific subgroups Provides a variety of metrics to track performance, such as reactions to and engagement with ads	Takes experience to get audience parameters correct Negative comments need frequent monitoring; negative comments and hate speech can have negative impacts on study staff as well as on potential study participants Not able to reach those not on Facebook Little to no direct customer service if ads were deemed to violate Facebook policy Takes significant staff time to optimize Learning platform requires time and research Requires thoughtful budgeting due to varied and somewhat unpredictable costs and efficacy of ads
Community outreach	Reach those not affiliated with the VA or connect with wider audience Can reach those not on social media Establish trustworthiness and credibility with leaders of community organizations who hold closer personal relationships with organization members	Little control to how or when organizations shared study information High-volume surges of interested participants to manage; surges may cause recruitment bias by geographic region or demographic group May be biased toward those involved with community groups (eg, those who publicly identify as LGBTQ+^b^ vs those who do not) Some organizations may be hard to reach or may not have up-to-date contact information Researching, compiling, contacting, and following up with organizations may require significant time and energy Some relevant local groups may be missed if study team is not connected or familiar with the region
Trialfacts or similar recruitment service company	Expertise and resources solely dedicated to recruitment and social media advertising Reduces burden on study staff Typically offers a recruitment number guarantee—will prorate a refund if numbers are not achieved Familiarity with institutional review board protocols Affordable or cost-efficient for this study Provides a tailored recruitment plan and study timeline	Can be expensive Less control over advertisements Requires establishing secure information exchanges with Trialfacts to meet all VA information security guidelines May lack content area expertise for specific populations, such as LGBTQ+ veterans Difficulty recruiting some subgroups Quality checks needed to review data or reports May overlap in reach if study is already conducting separate social media recruitment Not able to reach those not on social media

^a^VA: Veterans Health Administration.

^b^LGBTQ+: lesbian, gay, bisexual, transgender, queer, and other sexual and gender minority.

### Population Subgroups

Despite the overall success in recruiting LGBTQ+ veterans in this longitudinal study, there were areas where the recruitment underperformed. In particular, the number of bisexual veterans of any gender and transgender male veterans recruited was lower than the study aims. The reasons for underrecruitment likely differed between the groups.

Transgender men appear to be less prevalent in the veteran population compared with transgender women, although more accurate population estimates are needed [3,38]. This may be one reason why the study team had greater difficulty reaching a target goal of 200 for transgender men compared with other LGBTQ+ subgroups. In addition, transgender men face the barriers of stigma and invisibility [39,40]. For instance, a national study of transgender men found that 14.1% of participants were refused care by a provider due to their gender identity, and 32.8% delayed needed care due to fear of discrimination because of their gender identity [39]. These obstacles may reduce participation in health research.

Difficulties in recruiting bisexual veterans into research studies may be due to biphobia within and outside the LGBTQ+ community. Although bisexual people represent a large proportion of the LGBTQ+ community among both veterans and civilians [41], structural, interpersonal, and internalized biphobia may result in the exclusion of or decreased sense of belonging among bisexual people from the LGBTQ+ community [41,42]. This may make it difficult to recruit bisexual veterans through broadly targeted LGBTQ+ ads or through LGBTQ+ community groups. In addition, changes in sexual orientation identity labels over time also mean that veterans with same-gender or different-gender attractions do not necessarily identify as bisexual. Although the screening survey included other sexual identity options (eg, pansexual), the screener also required the selection of a single sexual orientation that best represented the interested participants’ sexual identity (choosing from 1 of the following: gay or lesbian, bisexual, or heterosexual) for study stratification purposes. Ultimately, improved methods for recruiting bisexual veterans and other veterans with both same-gender and different-gender partners in health research deserve further investigation. This is particularly important because the literature indicates that bisexual community members, especially bisexual women, experience health inequities in mental and physical health compared with lesbian and heterosexual women [43,44].

Notably, the study enrolled 66 gender-diverse veterans (a category that included nonbinary, gender nonconforming, and genderqueer veterans and veterans of other gender identities with any sexual orientation) despite not being an a priori recruitment category. This indicates a promising future area of research and engagement. There is limited research on the health of gender-diverse populations in general. Current literature shows that gender-diverse individuals face a number of inequities in health and social determinants of health, including increased risk of poverty, uninsurance, homelessness, discrimination in the health care system, suicide attempts, and psychological stress [45] compared with their cisgender peers.

Finally, racial and ethnic minority veterans were difficult to recruit across the subgroups in the study. The enrolled sample was less racially diverse compared with the overall veteran population, with 21.4% of the study sample identifying as racial and ethnic minority people compared with 26% in the wider veteran population [46]. Racial and ethnic minority veterans were intentionally included in targeted recruitment efforts, including through representation in ad images, outreach to community organizations specifically centering on racial and ethnic minority veterans, and Trialfacts’ advertising strategies. It is possible that a deeper community-based partnership building ahead of study recruitment would improve the perceived trustworthiness of the study and increase outreach to racial and ethnic minority veterans, particularly those within the LGBTQ+ community. Generally, the multiple layers of marginalization experienced by LGBTQ+ racial and ethnic minority veterans may add structural and interpersonal barriers. For example, racism within LGBTQ+ and veteran communities may also result in further marginalization of LGBTQ+ racial and ethnic minority veterans from both communities, resulting in fewer chances to hear about or less desire to participate in this study. In addition, recruitment for this study occurred during the COVID-19 pandemic and a wave of ongoing violence and racism against Asian American, Black American, and other racial and ethnic minority individuals. This could have made it more difficult for racial and ethnic minority veterans to hear about or participate in research studies, even with study compensation.

### Other Recruitment Challenges

It is impossible to know whether the COVID-19 pandemic increased or decreased participation in the study and for whom. Potentially eligible veterans may have been prevented from participating due to COVID-19–related stressors, such as physical, mental, emotional, and social health needs, as well as socioeconomic stressors such as lost work, housing instability, childcare responsibilities, financial uncertainty, and loss of access to internet or technology. By contrast, the entirely web-based nature of the *Health for Every Veteran Study* may have facilitated participation during a time of social distancing and stay-at-home orders, particularly compared with other research studies requiring in-person recruitment and enrollment.

Other factors that impacted the study recruitment process were as follows: (1) US congressional budget delays impacting VA research processes, (2) high-volume surges of heterosexual participants from community outreach, and (3) negative comments on Facebook ads. Congressional budget delays caused 2 pauses in social media advertising during the study recruitment period: from October 5, 2019, to October 25, 2019, and from October 1, 2020, to October 5, 2020.

On several occasions, the study team experienced high-volume surges of screeners from interested participants, which primarily occurred when conducting community outreach with large veterans’ organizations. This resulted in large numbers of heterosexual veterans completing the initial screener, which necessitated manual screening by staff to reduce bias in the sample toward one particular community organization (see the *Methods* section). Underrepresented subgroups in the sample were prioritized for enrollment, including women, LGBTQ+ subgroups, and racial and ethnic minority veterans. This may have introduced sampling bias, including for racial and ethnic minority, cisgender, and heterosexual veterans, who had a greater likelihood of being enrolled. However, this type of manual review was necessary to create a balanced study sample, reduce overall bias, and meet enrollment goals.

Finally, the study staff encountered a large volume of negative and derogatory comments on Facebook ads, up to dozens per day. These comments were predominantly anti-LGBTQ+ and often expressed opposition to the content of the ad images. Automatic filters allowed the study staff to hide comments immediately with certain keywords and phrases. In addition to these filters, the staff monitored the ad accounts throughout the day to manually hide the comments to minimize any potential negative impact on the LGBTQ+ study participants. Unfortunately, it is possible that some potential participants may have been exposed to and adversely affected by negative and derogatory comments before the study staff members were able to manually delete them. Filters and monitoring also served to discourage commenting in general, keeping recruitment in line with the VA institutional review board protocols, which do not allow direct social media interactions between the study team and veterans. Some ad images were adjusted based on constructive criticism shared in comments (most commonly, viewers criticized any images that altered the standard US military uniform). In addition, recognizing that health disparities research may attract scholars and study staff who hold one or more marginalized identities, it was important for the study team to build support mechanisms around social media monitoring. The study team regularly discussed recruitment topics in weekly meetings, which included conversations about the emotional and cognitive labor involved in monitoring derogatory Facebook comments. The team decided to rotate the task of moderating Facebook ads to ensure that a single person would not be overwhelmed by regular exposure to hate speech.

### Limitations and Strengths

There are limitations to this study, one of which is generalizability. To some extent, our processes and experiences were influenced by VA regulations, which reduces generalizability to non-VA researchers. Although we made concerted efforts to improve the national representativeness of our enrolled cohort, there were participant characteristics that we did not collect (eg, rurality) and could not be used to fine-tune our recruitment methods.

Importantly, our specific study design and subgroup stratification affected the recruitment methodology described in this paper. Because different recruitment strategies were used to attract different subgroups of participants, it was not possible to analyze which subgroups responded best to various types of recruitment methods. Consequently, it is difficult to draw concrete conclusions on whether specific methods are favored by certain subgroups. Overall, the nonprobability manner of recruitment is a significant limitation of this study. Therefore, the study results may not be generalizable to other populations. However, the lessons learned and general recruitment methodology are pertinent to a wide variety of research. In addition, our subgroup stratification still offers important disaggregated insights into the recruitment of specific LGBTQ+ subgroups.

The completely web-based methodology of the study served as both a strength and a limitation. Crucially, web-based recruitment allowed the recruitment to continue, even when the COVID-19 pandemic began. As such, this study offers tested recruitment options for other health research studies that will begin or continue recruitment during the pandemic or for other studies using solely web-based methods. However, the recruitment methodology could also have resulted in selection bias in the sample, as there are veterans who may not use social media and who may not be connected with community organizations. In addition, the social media platforms used may also have caused selection bias based on the user base (eg, age of users). However, our widespread community-based organizations targeted veterans across a variety of age ranges and other demographics.

Overall, this study contributes to the current literature on both LGBTQ+ and veteran health research recruitment. In the LGBTQ+ recruitment literature, this study offers insights into the recruitment of multiple LGBTQ subgroups. In addition, we add to the current literature on web-based recruitment through social media. Uniquely, we used community outreach in a completely web-based manner, offering opportunities for community-based recruitment efforts when face-to-face recruitment is not feasible. We add to the veteran recruitment literature by relying on methods that do not use VA administrative or EMR data. This improves the study’s generalizability by using recruitment methods that are inclusive of veterans outside the VA health care system.

### Conclusions

We share our descriptions and findings of the recruitment process of the *Health for Every Veteran Study* to convey the lessons learned during the web-based recruitment of LGBTQ+ veterans. As the first study to recruit a large and diverse group of veterans that varies across gender identity and sexual orientation, important insights were gathered that have implications for the web-based recruitment of LGBTQ+ veterans and potentially other hard-to-reach populations. The multipronged recruitment plan that consists of social media advertising, outreach to community organizations, and partnering with a recruitment company offers 3 viable options for researchers who plan to conduct web-based recruitment for health research studies.

## Abbreviations

CONSORT: Consolidated Standards of Reporting Trials
EMR: electronic medical record
IRB: Institutional Review Board
LGBTQ+: lesbian, gay, bisexual, transgender, queer, and other sexual and gender minority
VA: Veterans Health Administration
VCC: Veteran Care Coordinator

## Data Availability

The data sets generated and analyzed during this study are available from the corresponding author upon reasonable request.
